# Effectiveness of Teamwork in an Integrated Care Setting for Patients with COPD: Development and Testing of a Self-Evaluation Instrument for Interprofessional Teams

**DOI:** 10.5334/ijic.2454

**Published:** 2016-04-08

**Authors:** Anneke N. Van Dijk-de Vries, Inge G. P. Duimel-Peeters, Jean W. Muris, Geertjan J. Wesseling, George H. M. I. Beusmans, Hubertus J. M. Vrijhoef

**Affiliations:** Department of Family Medicine, CAPHRI School for Public Health and Primary Care, Maastricht University, Maastricht, The Netherlands; Department of Social Medicine, CAPHRI School for Public Health and Primary Care, Maastricht University, Maastricht, The Netherlands; Department of Family Medicine, CAPHRI School for Public Health and Primary Care, Maastricht University, Maastricht, The Netherlands; Department of Patient & Care, Maastricht University Medical Center, Maastricht, The Netherlands; Department of Family Medicine, CAPHRI School for Public Health and Primary Care, Maastricht University, Maastricht, The Netherlands; Department of Respiratory Medicine, CAPHRI School for Public Health and Primary Care, Maastricht University Medical Center, Maastricht, The Netherlands; Saw Swee Hock School of Public Health, National University Singapore, National University Health System, Singapore, Singapore; Department Patient & Care, Maastricht University Medical Center, Maastricht, The Netherlands; Department of Family Medicine and Chronic Care, Vrije Universiteit Brussels, Brussels, Belgium

**Keywords:** Teamwork, integrated care, self-evaluation, instrument, psychometric properties

## Abstract

**Introduction:**

Teamwork between healthcare providers is conditional for the delivery of integrated care. This study aimed to assess the usefulness of the conceptual framework Integrated Team Effectiveness Model for developing and testing of the Integrated Team Effectiveness Instrument.

**Theory and methods:**

Focus groups with healthcare providers in an integrated care setting for people with chronic obstructive pulmonary disease (COPD) were conducted to examine the recognisability of the conceptual framework and to explore critical success factors for collaborative COPD practice out of this framework. The resulting items were transposed into a pilot instrument. This was reviewed by expert opinion and completed 153 times by healthcare providers. The underlying structure and internal consistency of the instrument were verified by factor analysis and Cronbach’s alpha.

**Results:**

The conceptual framework turned out to be comprehensible for discussing teamwork effectiveness. The pilot instrument measures 25 relevant aspects of teamwork in integrated COPD care. Factor analysis suggested three reliable components: teamwork effectiveness, team processes and team psychosocial traits (Cronbach’s alpha between 0.76 and 0.81).

**Conclusions and discussion:**

The conceptual framework Integrated Team Effectiveness Model is relevant in developing a practical full-spectrum instrument to facilitate discussing teamwork effectiveness. The Integrated Team Effectiveness Instrument provides a well-founded basis to self-evaluate teamwork effectiveness in integrated COPD care by healthcare providers. Recommendations are provided for the improvement of the instrument.

## Introduction

The care of people with long-term conditions is universally recognised as a major challenge to health care services [1]. Chronic obstructive pulmonary disease (COPD) will be a leading cause of morbidity and mortality worldwide by 2020 [1]. COPD is defined as a preventable and treatable disease state, characterised by a progressive airflow limitation that is not fully reversible [2]. Improvements in quality of life, reduction in the rate of decline in lung function, and prevention and treatment of exacerbations involve a wide range of skills and activities, which can only be provided when different healthcare providers from both primary and secondary care work together with patients [3]. Innovative integrated care models are being explored in order to provide cost-effective chronic care to meet local needs [4,5,6].

Productive collaboration and coordination between healthcare providers in the delivery of integrated care have become essential requirements for the provision of high-quality care [5,6,7]. To deliver high-quality, evidence-based chronic care to those patients at greater risk, the presence of a multidisciplinary team was found to be associated with more comprehensive service provision [8,9].

For healthcare providers to work as a team, it is not enough to put them together and expect that they will automatically know how to work collaboratively [10,11,12]. Economic and historical political factors may affect the effectiveness of multidisciplinary teamwork as well as the interprofessional nature of the healthcare team [13,14]. Therefore, team effectiveness is a complex and multifaceted construct. Literature reviews and available instruments show the numerous dimensions and aspects of teamwork effectiveness, indicating the careful process of selecting an instrument to measure team effectiveness [11,12,13,14,15,16,17,18].

As indicated by Reeves et al. [17], among various models outlining the key elements that contribute to team effectiveness (i.e. how well a team functions), only the Integrated Team Effectiveness Model ‘has acknowledged the wider systems factors on teamwork’ (p. 50). Furthermore, according to Integrated Team Effectiveness Model, the effectiveness of a healthcare team depends on the determinants of the following domains: the sociopolitical context in which the team exists, the organisational context in which the team is doing its work, the task design, the team process and the psychosocial traits of the team. Integrated Team Effectiveness Model is an integrated framework that can be tailored to specific types of teams and settings [19]. As such, Integrated Team Effectiveness Model possesses important features when focussing on integrated care, as is the case in the current study.

This article outlines the process in developing and pilot testing a new instrument, the Integrated Teamwork Effectiveness Instrument, designed to measure healthcare team professional members’ perception of working collaboratively. This study aimed: (1) to assess the usefulness of the Integrated Team Effectiveness Model for developing a valid and practical instrument for self-evaluation of teamwork effectiveness by professionals and (2) to assess the content validity of Integrated Teamwork Effectiveness Instrument in measuring the perceived effectiveness of healthcare professionals working together in the care for people with COPD in the Maastricht region, the Netherlands.

## Theory and methods

### Theoretical background

In discussing the differences in how professional teams work, Reeves et al. made a distinction between teamwork, collaboration, coordination and networking [17]. They view teamwork as the most focussed form with high levels of interdependence, integration and shared responsibility. The other three approaches are increasingly broader activities with reducing levels of interdependence and so on. Notwithstanding the tentative nature of this typology, ‘it begins to indicate how different types of interprofessional teamwork can be considered contingent on the nature of responding to local need’ [17, p. 45]. Interprofessional teams in health and social care come into existence to ensure that professions can complete a care task, or a combination of tasks, that they could not achieve so effectively on their own [17].

Interprofessional teams vary in how well they function. In their summary of factors influencing team effectiveness, Reeves et al. pointed out that of models indicating factors that may influence team effectiveness, Integrated Team Effectiveness Model is the only framework considering factors at the systems level in addition to factors at the practice level and the organisational level [17].

Integrated Team Effectiveness Model is based on an extensive literature review [19] and uses Cohen and Bailey’s [20] team typology and team effectiveness model as a starting point for conceptualising healthcare team effectiveness. According to Cohen and Bailey, a multidisciplinary team can be defined as ‘a collection of individuals who are independent in their tasks, who share responsibility for outcomes, who see themselves and who are seen by others as an intact social entity embedded in one or more larger social systems and who manage their relationships across organisational boundaries’ [20]. Consequently, ‘teamwork’ is the product of interactions between healthcare professionals in a team.

Integrated Team Effectiveness Model depicts the complex interactions between task design, team processes, team psychosocial traits and team outcomes. Task design includes the type of team (e.g. project management and care delivery), task features (e.g. interdependence and autonomy), team composition (e.g. size and discipline) and organisational context (e.g. rewards and supervision). Task design factors can be manipulated by managers to improve team effectiveness. Team processes (e.g. communication and conflicts) are distinguished from team psychosocial traits (e.g. norms and shared mental modes). Both processes and traits are group-level phenomena that are influenced by task design and that can directly influence team outcomes; in addition, team processes and traits interact with each other. Task design can influence outcomes directly or indirectly [19]. Furthermore, the factors grouped under task design, team processed and team psychosocial traits play out on practice or team level, organisational level, and systems level and together determine the effectiveness of the teamwork [17].

Integrated Team Effectiveness Model distinguishes between objective and subjective outcomes. Objective outcomes include measurable improvements in patient outcomes (e.g. functional status and satisfaction), organisational outcomes (e.g. efficiency and costs) and staff behaviour (e.g. absenteeism and prescribing patterns). Subjective outcomes are attitudinal aspects of team effectiveness (e.g. team members’ perception of their teams’ effectiveness). All domains of Integrated Team Effectiveness Model are broad conceptual groupings that identify the essential indicators of team effectiveness in health care [19]. Distinguishing between objective (whether the goals of the team are achieved) and subjective outcomes (whether the team in itself works well together) is important considering that for the delivery of integrated care the formation of teams is still work in progress.

Notwithstanding the attractiveness of Integrated Team Effectiveness Model as a conceptual framework to the field of integrated care, it has not yet been translated into an instrument to be used by healthcare professionals to self-evaluate the effectiveness of their teamwork.

### Study setting

The integrated care setting for COPD patients in the Maastricht region includes one university hospital and 82 general practitioners working in 60 general practices. The multidisciplinary collaboration turned out to be a relevant issue for all COPD healthcare providers because a bundled payment system has been rolled out for COPD care in the Maastricht region. In brief, a bundled payment makes it possible for different elements of COPD care to be purchased, delivered and billed as a single product of service [21]. The services to be included have been described in a disease-specific healthcare standard. The development of the instrument was undertaken during the implementation phase of the bundled payment for COPD care; hence, the level of collaborative practice was variable between general practices. Nevertheless, participants were already familiar with the concepts of integrated care owing to a bundled payment for diabetes mellitus type 2 [21], joint consultations between the general practitioners and pulmonologists [22] and a chronic care programme for patients with asthma or COPD [23].

### Sample and procedure

The study was conducted in two phases. In phase 1 Integrated Team Effectiveness Model was applied to integrated COPD care and translated into a teamwork evaluation instrument. Phase 2 included an exploratory factor analysis for psychometric testing of the instrument.

#### Phase 1 – Instrument development

Conducting focus groups was the first step of instrument development [24,25]. The purposes of the focus groups were to examine the recognisability of Integrated Team Effectiveness Model and to explore all critical success factors for collaborative COPD practice based on this framework. Open-ended questions that covered the domains of Integrated Team Effectiveness Model [19] were used to initiate the conversation (see Table 1). A random sample of 26 general practitioners in the Maastricht region was invited to participate in the study. Due to perceived differences between collaborative practice in solo and groups practices [26], two focus groups met separately: general practitioners from solo and duo practices (*n* = 7) and general practitioners working in group practices (*n* = 4). Three out of four practice nurses, already involved in COPD care, volunteered to participate in a focus group. The focus group of respiratory nurse specialists consisted of four out of six participants. During the research project, one pulmonologist was involved in integrated care delivery, crossing the borders of primary and secondary care. This specialist was interviewed individually. All discussions were audio taped and transcribed verbatim.

**Table 1:** Items focus groups.

Domain Integrated Team Effectiveness Model	Question

Team effectiveness	What will effective teamwork in COPD care bring in and how can we measure that?
Organisational context	How do you define an organisational context with incentives for effective teamwork in COPD care?
Task design	How do you define your role in the COPD team?
Team processes	How do you define basic criteria for an effective team process considering the following key words: communication, collaboration, coordination, conflict, leadership, decision-making and participation?
Team psychosocial traits	What characteristics are required in a COPD team to be involved in the team and how important is that?

Following the focus groups, an analysis was carried out about primary indicators to be included in the instrument. Key stages followed were: familiarisation with the data, managing the data, mapping and interpretation [25]. The analysis was discussed among all members of the research team. In doing so, the generated topics in the concept instrument were compared with two literature reviews about teamwork to ensure that the items indicate determinants of effective collaborative practice [12,13]. Member check was accomplished by a multidisciplinary expert panel of COPD healthcare providers (*n* = 8). These healthcare providers were involved in the focus groups. The expert panel was asked to review the instrument; specifically, it was requested to analyse whether the items covered all the important areas and if the wording of the items was succinct. The panel also considered the practical use of the instrument. The feasibility of the instrument was verified by a pretest in one COPD team. This enabled the leading author to attend the teamwork evaluation dialogue guided by the instrument.

The Integrated Teamwork Effectiveness Instrument is oriented towards the healthcare providers’ perception of team-work effectiveness. For this reason, the indicators from the focus groups were reformulated into statements with answer choices on a 4-point Likert scale (ranging from strongly disagree to strongly agree). The items were phrased both positively and negatively. If a particular item did not apply, one could choose the answer ‘not applicable’. For facilitating discussion, respondents pointed out relevant issues for their team (‘important to discuss in our team: yes/no’ – for every particular item). Teams were asked about their ability to use the instrument without the presence of an outside consultant.

#### Phase 2 – Psychometric evaluation

Initial psychometric evaluation of the Integrated Teamwork Effectiveness Instrument included an exploratory factor analysis using the method of principal components [27]. The aim was to reduce data to a smaller set of components and to compare these dimensions with the underlying conceptual framework. In order to conduct a statistically meaningful factor analysis, it was aimed to include minimal 125 respondents to reach five subjects for 25 items [27].

First, all COPD practice nurses (*n* = 28; with exception of those in the team of the initial testing in phase 1) in the Maastricht region were requested to invite their COPD team for a teamwork self-assessment dialogue. They received the instrument with instructions by email. The team dialogue took place in the presence of at least a general practitioner and a practice nurse. Dependent on the level of teamwork in the general practice, practices nurses invited the nurse specialist, physiotherapist and/or dietician. The pulmonologist would complete the questionnaire, but for practical reasons he was not expected to attend all the team meetings during the pilot study.

To maximise the response and to enable psychometric testing of the instrument, individual core members of the COPD team (i.e. general practitioner, practice nurse, pulmonologist and respiratory nurse) were invited to complete the questionnaire without discussing it within their teams. Healthcare providers were requested to complete the questionnaire for the team(s) they were involved in.

## Data analysis

Data were analysed using SPPS version 15.0 (SPSS Inc., Chicago, IL). Given that the Integrated Team Effectiveness Model suggested different factors of team effectiveness to be dependent, a factor analysis with oblique rotation was applied to the 25 items. Tests of sampling adequacy (Kaiser–Meyer–Olkin (KMO)) and multicollinearity (Bartlett test of sphericity *p* < 0.05) were undertaken to check that the scale items were appropriate for factor analysis. The answer option ‘not applicable’ was treated as missing value. Pairwise deletion procedure was used to avoid extensive loss of responses due to single-item missing values. In total, there were 150 missing values. The number of missing cases on each variable is small relative to the sample size (1–20 missing values per variable, mean 6.0, ±6.1). Under listwise deletion technique, the sample size would have been *n* = 91.

The number of factors was assessed by Horn’s parallel analysis, which selects the factors that are greater than random [27]. Individual items were evaluated for inclusion or exclusion based on correlations, factor loading (greater than 0.35 [27,28]) and the logical interpretability of the item in relation to the extracted factor. For each scale emerging from the factor analysis, internal consistency using Cronbach’s alpha coefficients was calculated.

A Cronbach’s alpha score of 0.7 or higher is usually regarded as indicative of acceptable reliability [27,28].

## Results

### Phase 1 – Instrument development

The focus group members were able to translate every domain of Integrated Team Effectiveness Model into their daily practice. Each discipline stressed specific issues. The main differences between healthcare professionals relate to their scope of practice or their collaborative experience in COPD care. The indicators listed in Table 2 are the primary themes of teamwork effectiveness elicited by the interviews.

**Table 2:** Integrated Team Effectiveness Model applied to integrated care for people with COPD.

Domain Integrated Team Effectiveness Model	Focus groups

**Organisational context**	
Goals/standard	The collaborative practice aims to improve patients’ quality of life
Structure/characteristics	Clarity about structure and agreements of collaborative practice No competition among team members
Rewards/supervision	Team members are accessible for consultation
Training Environment	There are adequate training opportunities
Resources	The availability of time and work places
Information System	The information system is functioning and add relevant data
**Task design**	
Interdependence	Team members are interdependent to deliver quality of care
Autonomy	The input of every team member is valued
Clarity of rules and procedures	In general, the team will follow the care protocols.
**Team process**	
Communication	Relevant patient data are exchanged Team meetings are effective
Coordination	Communication contributes to continuity of care
Decision-making	A decision to be off track will be discussed within the team
Participation	Team members give priority to team meetings
Conflict	Open communication is valued
**Team psychosocial traits**	
Cohesion	Personal involvement in a COPD team
Norms	Mutual respect and trust between team members
**Team effectiveness**	
Objective outcomes	
Patient	Several indicators (e.g. exacerbation and quality of life) which are not only a determinant of teamwork
	Patient drop-out
Subjective outcomes	Patients know their primary contact person
Perceived team effectiveness	Satisfaction about the joint contribution to patients’ quality of life The care is patient-centred, not only disease specific The team has an overview about their patients

An expert panel of eight COPD healthcare professionals (three general practitioners, one pulmonologist, two practice nurses and two respiratory nurses) considered the instrument to be a useful and relevant checklist for discussing team effectiveness. They added one item about keeping an overall perspective of the person in addition to his/her COPD. The test panel was a COPD team including a general practitioner, practice nurse, two dieticians and two physiotherapists. They came to agreements about how to improve the registration and communication process. Their feedback led to revised procedures concerning the instrument. The final pilot instrument consisted of 25 items concerning organisational context, task design, team process, team psychosocial traits and team effectiveness. The word ‘team’ was found confusing for healthcare providers because: 1) they work in different ‘teams’ for different types of COPD patients and 2) they work on a (physical) distance of each other in various teams. For this reason it was decided to replace the word ‘team’ with ‘collaborative’ (in Dutch: samenwerkingsverband) to point out a group of healthcare providers that have a shared responsibility for the delivery of care to a population of patients with COPD.

### Phase 2 – Psychometric evaluation

Out of the 28 teams involving practice nurses, five of them organised a team evaluation meeting. These five teams brought in 21 questionnaires. In the second phase of data collection, 140 questionnaires were returned. Eight health-care providers did not complete the questionnaire (more than half of the items were missing) as they stated to lack experience working in the COPD team. The pulmonologist and three respiratory nurses only evaluated the COPD teams they were involved in (respectively 27 and 29 of 55 COPD teams). Eight practice nurses were working for two general practices. They evaluated the collaboration within these COPD teams separately. The final sample consisted of 153 questionnaires completed by 93 COPD healthcare providers (Table 3).

**Table 3:** Number of invited healthcare providers, response rate and number of returned questionnaires.

Discipline	Number of respondents	Response rate (%)	Number of questionnaires

General practitioner	53	65	53
Practice nurse	32	71	40
Pulmonologist	1	100	27
Respiratory nurse	3	60	29
Dietician	3	NA	3
Physiotherapist	1	NA	1
Total	93	NA	153

## Factor analysis

First, a factor analysis with oblique rotation was applied to the 25 items. For the total data set, KMO was equal to 0.81 and the Bartlett test was statistically significant (*p* < 0.001). It was decided to exclude one item (item 15) describing the effectiveness of team meetings. This item did not correlate with any other variable (*r* < 0.3) and the majority of p values were greater than 0.05. The item limited the interpretability of the underlying constructs and the reliability of the scales. Twenty-four items were left to be used in the factor analysis. The results are reported in Table 4. The average communality was 0.46 (± 0.10, range: 0.25–0.62).

**Table 4:** Communalities, component loadings of the PCA and Cronbach’s alpha.

Item	Cs*		Component		

			**1**	**2**	**3**

		**Component 1**			
		*In our COPD* ….			
4	0.51	… the electronic system for sharing data contributes to our teamwork	**0.75**	−0.01	−0.20
24	0.45	… The drop out of COPD patients is low	**0.67**	0.01	−0.02
25	0.44	… we not only focus on the disease but keep an overall picture of the patient	**0.63**	−0.21	0.17
23	0.39	… we have a better overview of our COPD patients due to our teamwork	**0.55**	0.00	0.17
22	0.42	… patients know their personal contact point	**0.51**	0.25	0.07
13	0.52	… we are providing patient data which are relevant for team members	**0.47**	0.27	−0.05
21	0.62	… I feel that we jointly are doing a good job in meeting patients’ care needs	**0.45**	0.21	0.41
9	0.25	… there are adequate opportunities to increase our knowledge about COPD	0.34	0.20	0.13
		**Cronbach’s alpha = 0.80 (7 or 8 items)**			
		**Component 2**			
		*In our COPD-team* ….			
5	0.59	… there is not enough available time for each other	−0.11	**0.81**	−0.21
2	0.54	… the different roles and responsibilities needs to be more clearly defined	−0.02	**0.72**	0.06
7	0.54	… the consultation function of team members needs to be more accessible	0.14	**0.68**	0.01
16	0.29	… we need more communication to provide continuity of care	0.31	**0.56**	−0.17
14	0.42	… team meetings are easily cancelled	−0.17	**0.55**	0.30
10	0.40	… we accomplish each other (vision, knowledge and skills)	0.21	**0.41**	0.23
12	0.48	… reasons to deviate from protocol are discussed	0.33	**0.38**	0.25
		**Cronbach’s alpha = 0.76 (7 items)**			
		**Component 3**			
		*In our COPD team* ….			
18	0.64	… team members feel that they are valued and respected	0.14	−0.20	**0.78**
17	0.51	… I know I can rely on the expertise of my team members	0.03	0.07	**0.68**
8	0.30	… competition interfered with my collaborative work	0.01	−0.10	**0.56**
19	0.52	… our communication is characterised by openness	0.37	−0.08	**0.54**
6	0.29	… a need for proper work rooms is impeding our team functioning	−0.27	0.12	**0.52**
1	0.45	… we are working towards a common purpose	0.29	0.03	**0.51**
20	0.36	… I consider myself as a member of this team	−0.01	0.30	**0.45**
11	0.54	… members of the team feel that their expertise is fully utilised	0.32	0.33	**0.38**
3	0.52	… we have well understood work agreements	0.30	0.34	**0.37**
		**Cronbach’s alpha = 0.81 (9 items)**			

The numbers in bold reflect a loading of items on either component 1, 2 or 3, where the factor loading is =>0.35. Cs*: communalities.

The factor analysis identified a 24-item questionnaire with three components of team effectiveness. The eigenvalues for the three components were 7.21, 2.06 and 1.68, respectively. These components explained 46% of the variance (30.06, 8.60 and 7.01, respectively). The first subscale measures different aspects of perceived team effectiveness. The second subscale involves in essence aspects of the team process. The third subscale mainly contains items of the teams’ psychosocial traits.

As we used a minimum cut-off of 0.35, item 9 about increasing knowledge did not sufficiently load on a component. Item 21, ‘I feel that we jointly are doing a good job in meeting patients’ care needs’, measures team effectiveness (0.45) and team psychosocial traits (0.41). Item 19, ‘our communication is characterised by openness’, is loading on team psychosocial traits (0.54) as well as team effectiveness (0.37). Cronbach’s alpha for the three components was 0.80, 0.76 and 0.81, respectively. The internal consistency of component 3 increased from Cronbach’s alpha = 0.81 to alpha = 0.82 by deletion of item 6.

## Discussion

The conceptual framework Integrated Team Effectiveness Model was found to be relevant in developing a practical full-spectrum instrument to self-evaluate teamwork effectiveness. Healthcare providers recognised the domains of Integrated Team Effectiveness Model and were able to apply them into their daily practice, although the adoption of multidisciplinary teamwork in COPD care was in its infancy during the study. The Integrated Teamwork Effectiveness Instrument provides a well-founded basis for discussing the effectiveness of teamwork in integrated care for people with COPD. Integrated Teamwork Effectiveness Instrument counts 24 items and 3 complementary subscales of team effectiveness with acceptable coefficients of reliability (Cronbach’s alpha ranging from 0.76 to 0.81). The sub-scales correspond to the following domains of Integrated Team Effectiveness Model: team effectiveness, team process and teams’ psychosocial traits. Due to the resentation of the statements (to what extent something affects teamwork), the subscales apparently are comprised of the items with regard to the organisational context and task design.

We purposefully chose Integrated Team Effectiveness Model to develop and test an instrument that facilitates discussion within a multidisciplinary team about the conditions and effectiveness of their teamwork. Integrated Team Effectiveness Model includes factors that influence team function and the ability of teams to perform effectively by obtaining clearly defined measures to questions at the practice, organisational and systems level [19]. Especially in the field of integrated care, this broad perspective seems highly relevant. Furthermore, Integrated Team Effectiveness Model dis-tinguishes between objective (whether the goals of the team are achieved) and subjective outcomes (whether the team in itself works well together). This is of relevance considering that for the delivery of integrated care the formation of teams is work in progress [19]. Nonetheless the attractiveness of Integrated Team Effectiveness Model, it is not exhaustive and may therefore miss out on factors or their interrelatedness in influencing team effectiveness. More research to test the (comparative) power of Integrated Team Effectiveness Model to explain team effectiveness is however needed. In the mean time, the present study has shed new light on the value of Integrated Team Effectiveness Model for healthcare professionals in self-evaluating their team effectiveness in the delivery of integrated care for people with COPD.

Although the teamwork assessment instrument was developed and tested for validity using mixed methods, this study encountered some methodological limitations. Integrated Team Effectiveness Model assumes that all factors contri-bute to team effectiveness. Nevertheless, there may be different levels of influence. The instrument is not able to assess factors individually or to grade the overall level of effectiveness of teamwork. We believe all items identified by healthcare professionals are important for teamwork. Therefore, each item (except for item 15 ‘the effectiveness of our team meetings is not sufficient’ due to statistical limitations) was included in the factor analysis in spite of low communalities. What is critical here is not the communality coefficient per se, but rather the extent to which the item plays a role in the interpretation of the factor.

In general, items that do not have a factor loading above 0.40 should be dropped from a scale and the instrument [28]. In the current study, the factor analysis aimed to sustain the usefulness of Integrated Team Effectiveness Model for developing a well-founded instrument. The purpose of the developed instrument was to provide a structured basis for a team discussion. For these reasons, a factor loading of 0.35 was regarded as being sufficient; thereby excluding item 9, ‘there are adequate opportunities to increase our knowledge about COPD’. This item does not directly relate to teamwork but rather is an indicator of the organisational context. We suggest reformulation of this item in ‘opportunities to increase our mutual knowledge about COPD’ in order to improve the connection with teamwork effectiveness.

The wording of other items also needs further improvements. Item 15, ‘the effectiveness of our team meetings is not sufficient’, is too vague. Based on the statistical analysis and interpretation, two questions are ambiguous as they are loading on different components. For item 21, ‘I feel that we jointly are doing a good job in meeting patients’ care needs’, the reason of loading on the team’s psychosocial traits might be the emphasis on ‘jointly’ that refers to a shared ambition. The double loading of item 19, ‘our communication is characterised by openness’ could be explained by the broad (Dutch) formulation.

Broad formulations were needed as the sociopolitical context in which COPD teams existed was in a state of flux; the memberships of both core and peripheral COPD teams were variable during this study. The focus groups were organised prior to the introduction of the bundled payment for COPD. This implied that changes in the task design domain were about to occur. For example, it was planned for practice nurses to take over certain tasks from respiratory nurses. If the interviews would have been held after the introduction phase, the interviews might have been more focussed, for example, about the collaboration between practice nurses and respiratory nurses. Furthermore, COPD teams were intensifying their cooperation with dieticians and physiotherapists. The contribution of these professions in developing the instrument would have been also valuable, though they were already able to apply the instrument to their practice. Thus, not only the perspective and experiences of healthcare providers determined the content of the teamwork instrument but also the broader context. This underlines the impact of the sociopolitical context with regard to team-work effectiveness [13,14,17].

In developing the Integrated Teamwork Effectiveness Instrument we generated the items and performed a confirmatory factor analysis in daily practice. For the latter, the sample size of this study was slightly below the recommended number of 200 independent observations. Furthermore, additional testing is necessary to assess other psychometric properties of the Integrated Teamwork Effectiveness Instrument including convergent, discriminant and criterion-related validity [24].

In this study, only the perspectives of the healthcare professionals were examined. As such, the instrument offered a starting point for a team performance interview with all healthcare providers involved. Customers of services delivered by a team (i.e. patients) should also participate in the assessment of teamwork effectiveness [16]. Existing instruments for the assessment of teamwork by patients exist in different healthcare and long-term care settings (e.g. [29]), whereas within the field of integrated care such assessment is, if performed at all, often limited to one or a handful of items as part of a more comprehensive instrument (e.g. PACIC [30]). Use of the Integrated Teamwork Effectiveness Instrument may be followed by the development, validation and use of an instrument for patients. Notwithstanding the importance of the patient as being a member of the team, an improvement of coordination and teamwork between pro-fessionals might already benefit the degree to which health care is experienced as coherent and connected and con-sistent with the patient’s needs and personal context [31].

The Integrated Teamwork Effectiveness Instrument assumes not to be an exhaustive numeration of determinants that affects teamwork effectiveness in COPD care, but it provides team members an instrument for a systematic self-evaluation and a basis for discussions about teamwork. This instrument was developed in the Dutch healthcare setting of integrated COPD care. We believe other teams in the Netherlands and other countries can further test the Integrated Teamwork Effectiveness Instrument. However, the instrument itself may need some contextual adaptation before being tested in other countries. In doing so, this study can serve as a resource to adapt the Integrated Team-work Effectiveness Instrument.

Although the checklist is positively evaluated by the expert panel, this study did not examine the actual use of the instrument in practice. Due to the timing of the second phase of this study (the bundled payment was just introduced), practice nurses were not ready for coordinating an evaluation of teamwork. Therefore, the key factors for successful implementation and the contribution as well as the responsiveness of Integrated Teamwork Effectiveness Instrument with regard to the improvement of teamwork effectiveness need further research. Feedback about team effectiveness may improve the motivation of team members and energises them for higher performance [16].

A better understanding and measuring of teamwork effectiveness is relevant for collaborative practice. In this study, the Integrated Teamwork Effectiveness Instrument was developed by using the Integrated Team Effectiveness Model. This theoretical model was found applicable to the experiences of healthcare professionals in COPD care. A practical assessment instrument that evaluates teamwork effectiveness could be derived from interviews based on the Integrated Team Effectiveness Model. Healthcare professionals perceived Integrated Teamwork Effectiveness Instrument as a helpful instrument to assess their collaborative practice and to structure a dialogue about teamwork. The factor analysis identified three reliable subscales of team effectiveness, which corresponded to domains of Integrated Team Effectiveness Model. This study indicated that the healthcare providers’ perspective on the effectiveness of their teamwork could be translated into a well-founded teamwork assessment instrument.

## Competing Interests

The authors declare that they have no competing interests.
